# Pancytopenia with Development of Persistent Neutropenia Secondary to COVID-19

**DOI:** 10.1155/2022/8739295

**Published:** 2022-05-11

**Authors:** Kathie Wu, Yvonne Dansoa, Priyanka Pathak

**Affiliations:** ^1^Geisinger Medical Center, 100 N Academy Ave, Danville 17822, PA, USA; ^2^Department of Hematology/Oncology, 100 N Academy Ave, Danville17822, PA, USA

## Abstract

Viral infections have long been linked to hematologic dysfunction. With the rapid spread of COVID-19, various hematologic manifestations have emerged. While there have been several reports of immune thrombocytopenic purpura from SARS-CoV-2, concurrent lymphopenia and anemia have sparse. We describe a case of COVID-induced pancytopenia that presented months after initial COVID infection that initially responded to IVIG and steroids, but now with persistent neutropenia.

## 1. Introduction

Pancytopenia describes a condition in which there are simultaneous decreases in all three cell lines and can be attributed to a number of causes including nutritional deficiency, medications, malignancy, autoimmune disease, and infection. Viruses especially have long been associated with hematologic dysfunction, and the emersion of COVID-19 proved no exception [1]. There have been reports of isolated cell lines being impacted by COVID [2, 3], but we present a unique case of late onset pancytopenia in an immunocompetent patient, complicated by the development of unresolved neutropenia secondary to COVID.

## 2. Case Presentation

A 64-year-old female, with the medical history significant for only a remote history of breast cancer that was treated with lumpectomy in 2010, presented initially to the primary care office with concerns of petechiae of her oral mucosa. A few months prior, she got routine testing for COVID through work and found she was positive for COVID but was asymptomatic at that time. Routine blood work was obtained which revealed significant leukopenia and thrombocytopenia with WBC of 1.82 and platelets less than 3. Patient was given 2 units of platelet transfusion and admitted to the hospital.

**Figure 1:**
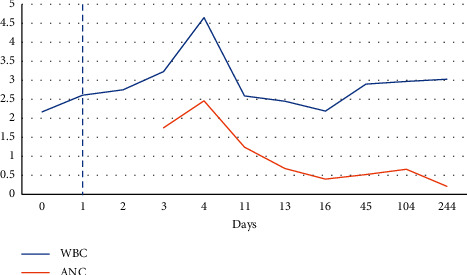
Trend in white blood cell count (WBC) in units of 10^9^ L. Dashed line represents administration of IVIG and steroid therapy.

**Figure 2:**
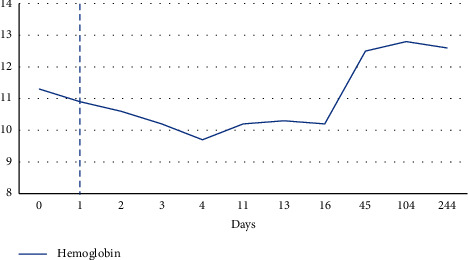
Trend in hemoglobin in units of g/dL. Dashed line represents administration of IVIG and steroid therapy.

**Figure 3:**
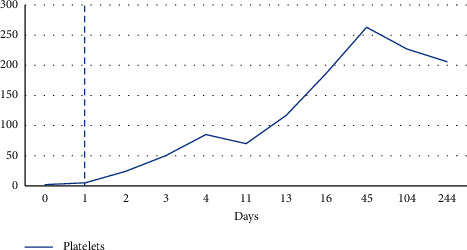
Trend in platelets in units of 10^4^ *μ*L. Dashed line represents administration of IVIG and steroid therapy.

On hospital day one, labs showed still worsening leukopenia with white count dropping further to 1.58. Despite the 2 units of platelet transfusion prior, platelet levels were still undetectable. Patient had no signs of active bleeding, but hemoglobin levels were still noted to drop by almost a gram from 11.8 to 10.9. Given high suspicion for an immune-mediated etiology of the pancytopenia, patient was started on dexamethasone 40 mg for 4 days and 2 doses of IVIG therapy. By hospital day four, patient continued to show rebound in her white count and platelet levels and her hemoglobin levels were starting to plateau off (Figures 1–3). Various tests were sent to determine underlying etiology of the pancytopenia. Laboratory tests including viral hepatitis, HIV, parvovirus, and rheumatological markers were unremarkable. Blood work was not consistent with a consumptive coagulopathy or thrombotic microangiopathy, and peripheral smear showed no blasts or schistocytes. A direct Coombs test, however, was positive with warm agglutinin, but the patient had nonelevated total or direct bilirubin levels at any point during her hospitalization that would have been suggestive of hemolytic anemia.

On one week follow-up, the patient had repeat blood work that showed sharp decline in both her white cell and platelet levels (Figure 1 and Figure 3). A bone marrow biopsy was pursued which revealed no concerning abnormalities. A few months later, blood work showed resolution of the anemia and thrombocytopenia, but patient was noted to have persistent leukopenia with ANC as low as 210 (Figure 3). As patient was otherwise asymptomatic, no further steroids or IVIG therapy was administered, but patient remains under close surveillance.

## 3. Discussion

Pancytopenia can result in the setting of nutrition deficiency, medications, or autoimmune processes, but with the improvement of the white count, hemoglobin, and platelets with IVIG and steroids, the positive Coombs, and considering a largely otherwise negative work up, the etiology of this patient's pancytopenia was thought to be immune mediated from her recent COVID infection. The effects of viral infections on hematopoiesis are well known, but the exact etiology by which viruses impact hematopoiesis are still under investigation. Various mechanisms including direct suppression of hematopoiesis by the virus, production of proinflammatory cytokines in response to viral infections, and invasion of the infection into the bone marrow have been hypothesized [4, 5]. Interestingly, there have been case reports published which have demonstrated infiltration of COVID-19 into the bone marrow that has tested positive via viral PCR [6].

COVID-19 causes a profound inflammatory response, and our patient initially responded well to steroid therapy to suppress inflammation which likely explains the initial improvement in her cell lines. As she only received four days of steroids which was a relatively short course, there may have been smoldering inflammation that was not adequately sequestered, allowing proinflammatory cytokines to damage the bone marrow. The initial bone marrow biopsy was unrevealing, but it may have been obtained early enough that the development of fibrosis was not yet detected. As our patient showed no infectious symptoms, there was no role for the use of granulocyte colony stimulating factor (G-CSF).

## 4. Conclusion

There have been emerging reports of a few cases of immune-mediated thrombocytopenia due to COVID-19, but literature has still been sparse, and most effects were seen in patients acutely hospitalized with COVID-19 infection, unlike our patient who presented months after initial resolution of the virus [7]. Again, there are many postulated mechanisms for etiology of disease, but what has been consistent across literature has been the response of COVID-19-induced pancytopenia to short courses of corticosteroids and intravenous immunoglobulin. Though there appears to be appropriate response to treatment, the progression of disease can be unpredictable as seen in our patient who initially improved with the same therapy then rebounded. The duration of the inflammatory effects of COVID-19 is unknown, but our patient's persistent neutropenia suggests that patients who have hematologic involvement during COVID-19 may benefit from longer courses of steroids. Though the bone marrow biopsy did not formally show bone marrow fibrosis, a longer course of steroids may help to reduce the activity of proinflammatory cytokines for longer periods of time and diminish possible long-term damage to the bone marrow. This case indicates that we may see lingering effects of COVID-19 well beyond the pulmonary manifestations of the disease and active surveillance must be maintained.

## Data Availability

The data used to support the findings of this study are included within the article.
